# What Is Intensity and How Can It Benefit Exercise Intervention in People With Stroke? A Rapid Review

**DOI:** 10.3389/fresc.2021.722668

**Published:** 2021-09-21

**Authors:** Gavin Church, Christine Smith, Ali Ali, Karen Sage

**Affiliations:** ^1^Community Stroke Service, Sheffield Teaching Hospitals National Health Service Foundation Trust, National Institute of Health Research Pre Doctoral Fellow, Sheffield Hallam University, Sheffield, United Kingdom; ^2^Department of Allied Health Professions, Advanced Wellbeing Research Centre, Sheffield Hallam University, Sheffield, United Kingdom; ^3^Stroke Consultant and Stroke Research Lead, National Institute of Health Research Biomedical Research Centre, Sheffield Teaching Hospital, Sheffield, United Kingdom; ^4^Faculty of Health, Psychology and Social Care, Manchester Metropolitan University, Manchester, United Kingdom

**Keywords:** stroke, exercise prescription, intensity, outcomes, international classification of function

## Abstract

**Background:** Stroke is one of the major causes of chronic physical disability in the United Kingdom, typically characterized by unilateral weakness and a loss of muscle power and movement coordination. When combined with pre-existing comorbidities such as cardiac disease and diabetes, it results in reductions in cardiovascular (CV) fitness, physical activity levels, functional capacity, and levels of independent living. High-intensity training protocols have shown promising improvements in fitness and function for people with stroke (PwS). However, it remains unclear how intensity is defined, measured, and prescribed in this population. Further, we do not know what the optimal outcome measures are to capture the benefits of intensive exercise.

**Aim:** To understand how intensity is defined and calibrated in the stroke exercise literature to date and how the benefits of high-intensity training in PwS are measured.

**Methods:** A rapid review of the literature was undertaken to provide an evidence synthesis that would provide more timely information for decision-making (compared with a standard systematic review). Electronic databases were searched (including Medline, PubMed, CINAHL, and Embase for studies from 2015 to 2020). These were screened by title and abstract for inclusion if they: (a) were specific to adult PwS; and (b) were high-intensity exercise interventions. Eligible studies were critically appraised using the Mixed Method Appraisal Tool (MMAT). The data extraction tool recorded the definition of intensity, methods used to measure and progress intensity within sessions, and the outcomes measure used to capture the effects of the exercise intervention.

**Results:** Seventeen studies were selected for review, 15 primary research studies and two literature reviews. Sixteen of the 17 studies were of high quality. Nine of the primary research studies used bodyweight-supported treadmills to achieve the high-intensity training threshold, four used static exercise bikes, and two used isometric arm strengthening. Five of the primary research studies had the aim of increasing walking speed, five aimed to increase CV fitness, three aimed to improve electroencephalogram (EEG) measured cortical evoked potentials and corticospinal excitability, and two investigated any changes in muscle strength. Although only one study gave a clear definition of intensity, all studies clearly defined the high-intensity protocol used, with most (15 out of 17 studies) clearly describing threshold periods of high-intensity activity, followed by rest or active recovery periods (of varying times). All of the studies reviewed used outcomes specific to body structure and function (International Classification of Functioning, Disability, and Health (ICF) constructs), with fewer including outcomes relating to activity and only three outcomes relating to participation. The reported effect of high-intensity training on PwS was promising, however, the underlying impact on neurological, musculoskeletal, and CV systems was not clearly specified.

**Conclusions:** There is a clear lack of definition and understanding about intensity and how thresholds of intensity in this population are used as an intervention. There is also an inconsistency about the most appropriate methods to assess and provide a training protocol based on that assessment. It remains unclear if high-intensity training impacts the desired body system, given the diverse presentation of PwS, from a neuromuscular, CV, functional, and psychosocial perspective. Future work needs to establish a clearer understanding of intensity and the impact of exercise training on multiple body systems in PwS. Further understanding into the appropriate assessment tools to enable appropriate prescription of intensity in exercise intervention is required. Outcomes need to capture measures specific not only to the body system, but also level of function and desired goals of individuals.

## Introduction

Worldwide, 15 million people suffer strokes each year of which 5 million die and another 5 million are permanently disabled (1). Within the United Kingdom, a stroke occurs in individuals every 5 minutes, affecting over 100,000 people each year, who join a population of 1.2 million people with stroke (PwS) (2). Stroke remains the fourth highest cause of death in the United Kingdom after dementia, ischemic heart disease, and respiratory disease.

A stroke occurs when there is a sudden insult to the central neurological system because the blood supply to the brain is impeded. It can lead to a number of physical, cognitive, and psychological difficulties. Severe hemiplegia presenting as unilateral paralysis of the arm and leg is the most common physical symptom, which in 57.7% of cases affects the right side of the body (3), with the upper limb being more severely involved due to the high proportion of strokes involving the middle cerebral artery (4). One week after the stroke, hemiplegia is still present in 89.1% of PwS, while at 1 month, 72% of individuals continue to experience unilateral weakness or hemiparesis (5). After 6 months, the incidence of hemiparesis is observed in at least 65% of PwS (6, 7). This paresis results in an inability to generate muscle strength that leads to abnormal posture, abnormal stretch reflex, reduced power production, and impaired voluntary movement (6).

PwS commonly present with pre-existing comorbidities that are already likely to compromise their CV function and fitness. PwS and those experiencing myocardial infarction demonstrate similar characteristics in relation to age of onset and prevalence of hypertension, hypercholesterolemia, heart failure, diabetes, and peripheral vascular disease (8, 9). Similar patterns are found in individuals presenting with diabetes as a comorbidity, resulting in changes to insulin resistance and changes to blood cellular biochemistry including the role of glucose transporter 4 (GLUT4) in facilitating glucose uptake to maintain control of blood glucose levels (10). These risk factors result in changes to CV fitness at rest and during submaximal exercise. This includes a reduced or preserved cardiac ejection fraction with reduced cardiac output, a reduced stroke volume, increased difference in arterial-venous oxygen levels, increased total systemic vascular resistance, reduced skeletal muscle mitochondrial density, and reduced skeletal muscle oxidative capacity (10–12).

These primary impairments post-stroke combined with potential comorbidities result in a further reduction of functional capacity through effects on metabolic function, immune and hormonal profile, and bone mineral density (12). Furthermore, this gives rise to a moderate to strong correlation with functional performance and gait velocity (13), with some authors reporting how a pathological gait of hemiplegia may have double energy costs compared to those of a healthy subject (14).

Exercise and physical activity play an important role in preventing and managing health conditions such as coronary heart disease, type 2 diabetes, stroke, mental health problems, musculoskeletal conditions, and some cancers. It also has a positive effect on well-being and mood, providing a sense of achievement or relaxation and release from daily stress (15). Physical activity has been defined as any bodily movement produced by skeletal muscles that requires energy expenditure. This may be playing, working, active transportation, house chores, and recreational activities (16). Social changes over the last 40 years and the impact of disabling disease are among the biggest factors affecting physical activity levels (17). This has resulted in the need for supplementary exercise when physical activity levels are low, with the Department of Health suggesting that where 150 minutes of moderate exercise is not feasible, then 75 minutes of vigorous intensity activity, shorter durations of very vigorous intensity activity, or a combination of moderate, vigorous, and very vigorous intensity activity should be used instead (18).

Exercise has become a long-term rehabilitation strategy for PwS where a combination of strength training and aerobic training has been demonstrated to increase functional capacity in day-to-day living (19). The impact of exercise can be captured using several outcome measures (20). The WHO International Classification Functioning, Disability and Health (ICF) (21) is a dynamic multidimensional classification of health and health-related domains. It considers: (1) body functions and structure (aspects of anatomy and physiology); (2) activities (actions and tasks undertaken by an individual); (3) participation (involvement in real-life situations); and (4) the environment and personal factors that may influence an individual. Within the healthy population, changes as a result of exercise intervention are usually captured by looking for changes to body structure and function, i.e., changes to ventilatory threshold and cardiac functioning. More recently, exploring the use of the WHO ICF (21) outcomes in clinical groups, has demonstrated a similar picture with a focus on outcomes of body structure and function and fewer outcomes focusing on activity and participation levels (22).

Elmahgoub et al. (23) set out how CV exercise occurs over three different intensity levels, low, moderate, and vigorous, which are measured by the Metabolic Equivalent of Task (MET). The effects of exercise at each intensity level result in a different training effect, with changes to VO_2_, blood pressure (BP), blood lipid profiles, body mass index (BMI), blood glucose, mood, and quality of life (24).

In clinical practice, the percentage of maximal heart rate (MHR) or ventilatory threshold (VO_2_) is commonly used to measure the intensity of CV training. Studies of healthy individuals have commonly used an intensity range from 70 to 95% of a MHR, ventilatory threshold, strength, or lactate threshold to achieve fitness changes. It remains unclear if using a lower range of 30–85% of MHR as an intensity guide yields proportionally lower changes to an individual (24, 25).

Higher intensity training has gained much popularity in the last 10 years due to the short-term benefits to walking speed, CV fitness, muscle strength, and changes to health biomarkers. Laursen et al. (26) identified changes in muscle enzyme activity in highly trained athletes, following high-intensity training. Despite no change in oxidative or glycolytic enzyme activity, there were significant improvements in endurance performance (*p* < 0.05). They also identified how increases in skeletal muscle buffering capacity may be one of the mechanisms responsible for an improvement in endurance performance. Changes in plasma volume, stroke volume, as well as myoglobin, capillary density, and muscle fiber characteristics have yet to be investigated in higher intensity training.

Mangine et al. (27) and Schoenfeld et al. (28) explored physiological changes to muscle physiology and structure with high-intensity strength training. Strength-focused training typically does not use MHR or VO_2_ as a guide for intensity. Both strength-focused training and CV training rely on using a percentage of maximal power of an individual or strength production as a guide and working at a specific threshold of maximal intensity. Mangine et al. (27) and Schoenfeld et al. (28) identified changes to cross-sectional area, fiber type and size, pennation angles, and collagen content when comparing higher intensity with lower intensity training. They concluded that these changes are the most likely mechanism for improvement to fitness when compared with lower intensity training, despite both forms of training giving similar changes to metabolic functioning.

Optimal neuroplastic changes require a combination of skill, aerobic, and strength-based training to influence changes at cortical, subcortical, spinal, and peripheral levels of the nervous system (29). During neurological training, increasing the intensity of interventions appears to be one of the most beneficial components to improving functional performance (30). However, the definition of intensity, the aims of delivery, and the measurement of intensity in neurological or skills training is poorly understood and poorly standardized, when compared with CV and strength training (31–35).

It remains unclear if the underlying anatomical and physiological changes occurring during exercise as part of rehabilitation intervention at higher intensity positively affect all components of the WHO ICF framework. It also remains unclear if changes to outcomes to body structure and function (impairment), activities (limitations), or participation (restrictions) are affected, and if so, how is this captured in PwS (36).

Rapid reviews were introduced (37) to overcome a key barrier to the use of research evidence in decision-making (namely the delay in practitioners accessing and using research syntheses). In order to make the review rapid and timely, it restricts itself to studies that had been published recently (the last 5 years for example), excludes non-peer-reviewed work and unpublished/grey literature as well as avoiding non-English texts. A rapid review typically uses one reviewer only and has an optional quality assessment step (37, 38).

To date, there have been two systematic reviews covering high-intensity exercise for PwS (39, 40), which looked solely at what exercises were used in high-intensity training. Neither of these gave a clear definition for intensity and did not explore the aims of the intervention. This review therefore intends to fill these gaps in the exploration of intensity. The aims of this review are set out below:

1. Explore how intensity is defined within the exercise interventions for PwS.
2. Document the aims of the exercise interventions for PwS (e.g., cardiovascular function, muscle strength etc.).
3. Identify the methods/tools used to measure intensity during the exercise interventions.
4. Document how studies prescribe intensity in the exercise interventions for PwS and how intensity is monitored during exercise.
5. Identify outcome measures used to capture change as a result of exercise training in stroke and whether these are mapped across the WHO ICF constructs.

## Methodology

This study used guidance on the methodological process for a rapid review from Haby et al. (37) and Dobbins (38). The rapid reviews involve one reviewer and use strict eligibility criteria when selecting articles.

The sequential steps for this review are based on the Search, Appraisal, Synthesis and Analysis (SALSA) elements (41):

- Systematically search the literature and identify appropriate papers for the rapid review.
- Appraise the quality of papers using an appropriate quality assessment tool.
- Synthesize the content to identify themes and patterns.

The eligibility criteria followed those of Haby et al. (37) and Dobbins (38) and can be viewed in Table 1.

**Table 1:** Eligibility criteria for inclusion and exclusion and their justification.

**Code**	**Inclusion**	**Exclusion**	**Justification for rapid review Haby et al. (37) and Dobbins (38)**
1	Peer-reviewed literature from 2015 onwards	Peer-reviewed papers prior to 2015 Unpublished/draft publications Grey literature	Ensure up to date literature is reviewed and excludes literature that has not undergone peer review.
2	Intensity-specific exercise intervention	Intensity not part of the intervention	Intensity only literature
		Non-exercise specific	
3	Describes method used to deliver intensity	No description of the method used to deliver intensity	Intensity delivery methods must be identified.
4	Stroke specific clinical group	Non-stroke population (health/other clinical groups)	Review is specific to PwS and therefore other clinical groups and non-clinical groups have been excluded.
		Stroke data cannot be disaggregated from other clinical populations	
5	Participants 18 and over	Participants under 18	Excludes participants under 18 where physiological response to exercise may differ.
6	Human studies	Not involving humans	Ensures findings are generalizable to human participants.
7	Articles written in English	Non-English articles	Avoids translation time and costs needed for foreign studies.

Four databases (Medline, Pubmed, CINAHL and Embase) were searched in November 2020. Searches were restricted from January 2015 to November 2020.

A building block approach (42) identified search terms for each concept. The concepts were: exercise (Concept A); stroke (Concept B), and intensity (Concept C). The search strategy comprised:

(a) Terms to describe stroke
(b) Terms to describe exercise
(c) Terms to describe intensity

These are shown in Table 2.

**Table 2:** Example of search strategy including concepts, key words and MeSH terms.

**Concept A**		**Concept B**		**Concept C**
**MeSH Subject heading**		**MeSH Subject heading**		**MeSh Subject heading**
MeSH “Exercise+ or Activity+”		MeSh “Stroke+ or Cerebral Haemorrhage+” Stroke* or CVA or cerebrovascula* acciden*		MeSH “Intensity or intense”
**OR**		**OR**		**OR**
**Keywords**		**Keywords**		**Keywords**
Physica* activ* or physical exert* or exercis* therap*		post stroke or cerebrovascular or cerebral hemorrhage or cerebral vascula*		High-intensity or High-intensity interval training or HIIT or Moderate intensity interval training or MIIT
**OR (specific terms for types of exercise)**		**OR (result of or impact outcomes of stroke)**		**OR (Less commonly used)**
Exercise* or train* or strength* or strength* or isometric* or aerobic*. or endurance* or weigh* resist* or train or run*or job*. or walk*. or resistance* train* or Program*		TIA or transient isch* or infarct*or brain isch?emi* or aphasi*, Heminopia, Cognition?		
**Search set A**	**AND**	**Search set B**	**AND**	**Search set C**

MeSH, keyword, and specific term searches were completed. The Boolean operators AND and OR were to be used, alongside phrase, proximity, and truncation operators dependent on the database used. The search syntax was adapted for each information source and controlled vocabulary terms used where available.

Screening of papers on title and abstract was undertaken by the first author to identify those that met the inclusion and exclusion criteria. The first author then excluded papers by reading the full text. Ten of the papers excluded were sent to a second author (KS) for verification.

The extraction tool was developed and piloted by the first author (GC) on 10% of the papers. These were then checked and agreed by a second reviewer (KS).

The extracted data included basic information (authors, year of publication, type of paper, and location). In addition, more specific information to achieve the review outcomes included finding:

- Definitions of intensity
- Which body system the exercise was aimed at (e.g., CV system, muscular system, or neurological system)
- Measurement tools used in the assessment of intensity [maximal ventilation (VO_2_ max)/gas exchanges testing, rate of perceived exertion (RPE), HR, repetition maximum, functional outcomes, and patient reported outcome measures (PROM)]
- How intensity is prescribed in PwS
- Outcome measures used to quantify the effect of exercise at various intensities on the body (resting HR and BP, blood lipid profiles, VO_2_ max, 6-min walk test (6 MWT), shuttle run test, etc.)

Full-text articles identified as eligible during screening were then assessed for quality using the Mixed Methods Appraisal Tool (MMAT) (43). Papers were not excluded on the basis of the quality assessment. The quality assessment of studies provided an indicator of the robustness of the studies included in the review.

Narrative synthesis, with supporting tabular synthesis, drew together the information on:

- Homogeneity or heterogeneity in the terms used to define intensity within papers collected.
- Methods used in the testing and assessing fitness in PwS.
- Clarity of exercise-intensity prescription when used as an intervention and rehabilitation technique.
- How intensity impacts on changes to outcomes and what outcome measures within the constructs of the WHO ICF framework were commonly used.

## Results

**Figure 1:**
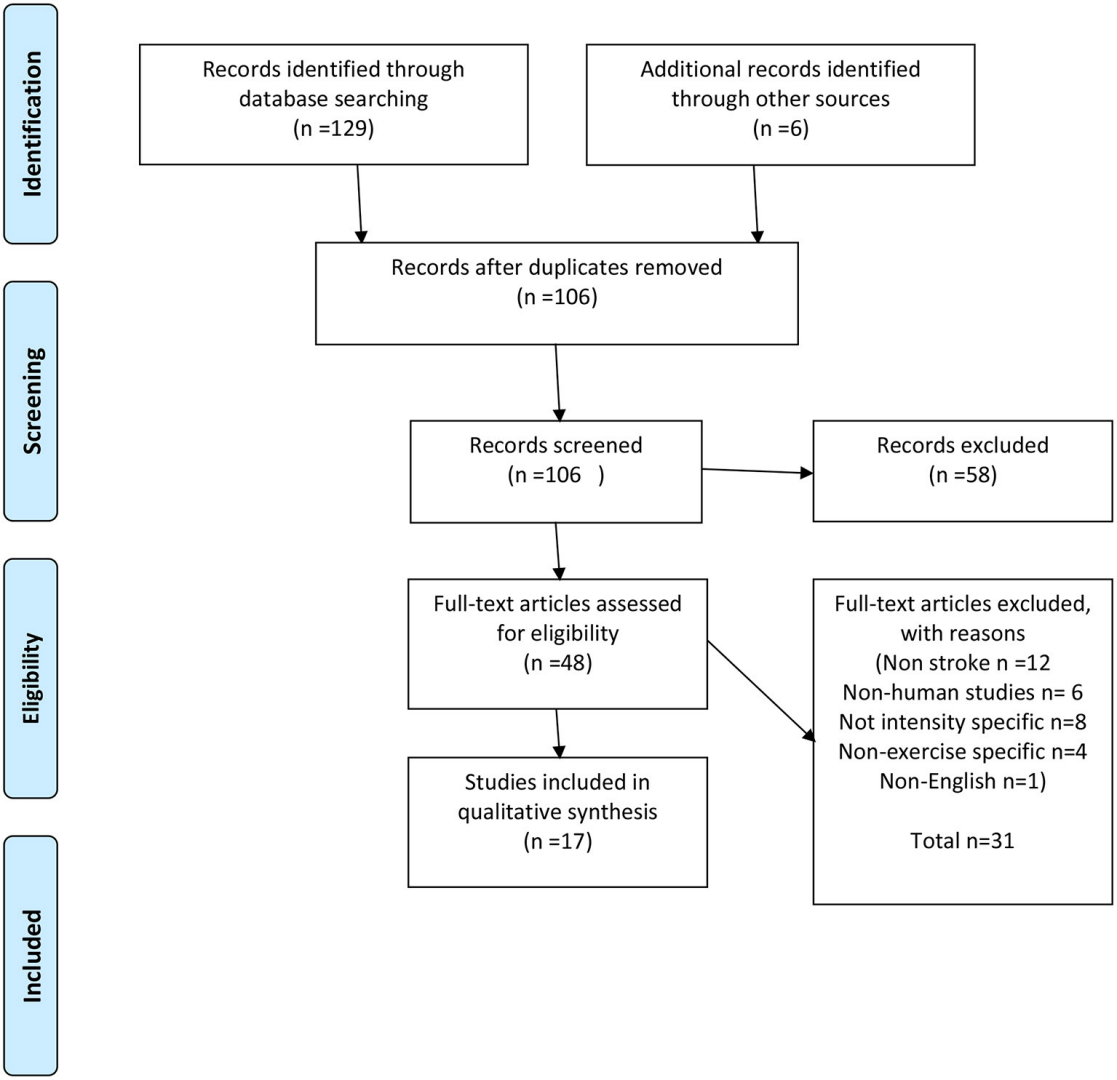
Prisma flow diagram.

Database searches found 129 records with an additional six from a reference list review. After duplicates were removed, 106 records were screened and 48 were screened by title and abstract for full-text eligibility assessment, leaving 17 articles for the purpose of this review. Of the articles excluded, 12 were non-stroke-related, 6 did not involve humans, 8 were not intensity-specific, 4 were non-exercise-specific, and 1 was only available in Chinese. A full breakdown of this process is included in Figure 1 (PRISMA flow diagram).

The 17 articles were subjected to MMAT evaluation; eight were quantitative non-randomized controlled trials (RCTs), eight were quantitative RCTs, and two were qualitative reviews of the literature. Eleven papers came from the Unites States, two from Canada, and single records from Norway, Germany, Denmark, China, and Italy. Participant numbers ranged from 6 to 36 in the primary studies reviewed.

The MMAT quality assessment indicated that all the articles had clear research questions, appropriate and clear data collection methods to address the question and approach, interpretation of the data, and coherence during the analysis and synthesis. Only one of the RCTs (44) failed to identify if assessors were blinded for the process.

Table 3 summarizes the findings from the included studies.

**Table 3:** Summary results of included papers: type of study, training effect, methods for increasing intensity.

**First name author**	**Paper title**	**Journal**	**Date**	**Location**	Type of study^*^	**Intended training effect**	**Method of assessing intensity**	**Method of monitoring intensity within session**	**Methods to progress intensity**
Aaron et al.	Feasibility of single session high-intensity training utilising speed and active recovery to push beyond standard practice.	Topics in Stroke Research	2018	USA	Quant Non-RCT	Walking speed	Walking speed on treadmill	Walking speed and quality	Incremental walking speed
Abraha et al.	A bout of high-intensity interval training lengthened nerve conduction latency to the non-exercised limb in chronic stroke.	Frontiers in Physiology	2018	Canada	RCT	Cardiovascular (CV) fitness, strength, upper limb function and cognitive timing	Maximum (VO_2_) max testing	%VO_2_ max	Increasing %VO_2_ and walking gradient
Boyne et al.	Within-session responses to high-intensity interval training in chronic stroke.	Clinical Sciences	2015	USA	Quant Non-RCT	CV fitness and walking speed	(GXT) for MHR for MHR and VO_2_	Walking speed and % maximal effort from % of GXT	Increasing walking speed and gradient
Carl et al.	Preliminary safety analysis of High-intensity interval training (HIIT) in persons with chronic stroke.	Applied physiology, Nutrition and Metabolism	2016	USA	Quant non-RCT	Safety	GXT for MHR and VO_2_	ECG	Reduced recovery times
Crozier et al.	High-intensity interval training after stroke: an opportunity to promote functional recovery, cardiovascular health and neuroplasticity.	Neurorehabilitation and Neural Repair	2018	Canada	Qualitative review	CV fitness and walking speed	VO_2_ max testing and walking speed	N/R	Variation of increasing %VO_2_ max, walking speed, recovery time
Gjellesvik et al.	Effects of high-intensity interval training after stroke (The HIIT stroke study)	Archives of physical medicine and rehabilitation	2020	Norway	RCT	CV fitness	VO_2_ max testing	% MHR	Increasing walking speed and gradient
Högg et al.	High-intensity arm resistance training does not lead to better outcomes that low intensity resistance training in patients after sub-acute stroke	Journal of rehabilitation medicine	2020	Germany	RCT	Upper limb strength and function	1 Repetition Maximal (RM) functional strength testing for upper limb	Range of motion and repetitions completed	Increasing range of motion and repetition until achieving 15
Krawcyk et al.	Effect of home-based high-intensity interval training in patients with lacunar stroke.	Frontiers in Neurology	2019	Denmark	RCT	CV fitness, meatal health and well-being, Body mass index and activity levels	Talk testing	RPE (BORG 6-20)	RPE (BORG 6-20)
Leddy et al.	Alterations in aerobic exercise performance and gait economy following high-intensity dynamic stepping training in persons with sub-acute stroke.	Journal neurological physical therapy	2016	USA	RCT	CV fitness and walking speed.	GXT testing for MHR and VO_2_	%MHR, gait quality, RPE (BORG 6-20)	%MHR and RPE (BORG 6-20)
Li et al.	A short bout of high-intensity exercise alters ipsilesional motor cortical excitability post stroke.	Topics in Stroke Rehabilitation	2019	USA	Quant non-RCT	Brain activity	Age predicted calculated MHR	%MHR and RPE (BORG 6-20)	Progressive walking speed to achieve target %MHR
Luo et al.	Effects of high-intensity exercise on cardiovascular fitness in stroke survivors.	Annals of Physical and rehabilitation medicine	2020	China	Qualitative review	CV fitness and walking speed.	Not discussed	N/R	N/R
Madhavan et al.	Effects of single session of high-intensity interval treadmill training on cortical excitability following stroke.	Journal of neural plasticity	2016	USA	Quant non-RCT	Brain activity and walking speed.	10-meter times walk	%MHR, RPE (BORG 6-20), blood pressure	10% increase in walking speed if able to tolerate previous session
Madhaven et al.	Effects of High-intensity speed-based treadmill training on ambulatory function in people with chronic stroke: A preliminary study with long term follow up.	Scientific Reports	2018	USA	Quant Non-RCT	Walking speed	Age predicted calculated MHR and 10-meter walk test	%MHR, RPE (BORG 6-20) and gait quality	Progressive increase from 50% walking speed until exceeding 80% MHR or gait disturbance
Mahtani et al.	Altered sagittal and frontal plane kinematics following high-intensity stepping training versus conventional interventions in sub-acute stroke.	Physical Therapy	2017	USA	RCT	Walking quality of movement	Age predicted calculated MHR and RPE (BORG 6-20)	RPE (BORG 6-20), %MHR and BP	%MHR and RPE (BORG 6-20)
Munari et al.	High-intensity treadmill training improves gait ability, VO_2_ and cost of walking in stroke survivors: preliminary results of a pilot RCT.	European Journal of Physical and Rehabilitation Medicine	2018	Italy	RCT	Walking quality of movement	Age predicted calculated MHR and Borg 6-20 PRE	%MHR and RPE (BORG 6-20)	%MHR and RPE (BORG 6-20)
Nepveu et al.	A single bout of High-intensity Interval training improved motor skill retention in individuals with stroke.	Neurorehabilitation and Neural Repair	2017	USA	Quant non-RCT	Brain activity.	GXT for MHR and VO_2_ with age predicted MHR	%MHR and RPE (BORG 6-20)	Participants working at 100% maximal walking speed- no progressions made
Urbin et al.	High-intensity unilateral resistance training of a non-paretic muscle group increases active range of motion in severely paretic upper extremity muscle group after stroke.	Frontiers in Neurology	2015	USA	Quant non RCT	Brain activity, strength and range of motion	1RM for isometric resistance strength	ROM and observed fatigued onset	Increasing range of motion and %1RM

* *Using MMAT definition*.

*N/R, Not reported; quant, quantitative; RCT, Randomized Control Trail; GXT, Graded Exercise Testing; RPE, Rate of Perceived Exertion; MHR, Maximal Heart Rate; VO_2_, Ventilatory oxygen threshold; RM, Repetition Maximum; BP, Blood pressure; CV, cardiovascular*.

### Homogeneity and Heterogeneity

The definition of intensity was only identified in one paper as “the work rate, effort level, or metabolic demand of aerobic activity quantified by heart rate, rate of oxygen consumption, rating of perceived exertion and/or walking speed” (45).

Collectively, the reviewed studies identified working at or above 80–90% of MHR, VO_2_, or one repetition maximum of an individual classifies as a high-intensity intervention, where moderate intensity was aimed toward 40–60% of these physiological outcomes. This does vary between studies and does not always consider rest intervals or ratio as used in Boyne et al. (44). The studies found by this review all focused on higher intensity exercise interventions. This may reflect the current trend in researching potential health benefits of higher intensity exercise for clinical and non-clinical groups when compared with lower intensity training (27, 28).

All of the primary research studies identified clear objectives for how they used high-intensity training protocols. Munari et al. (45) was the only study that discussed and defined intensity and the impact of intensity on participants. Neither review study (39, 40) provided definitions of intensity but shared similar findings to the primary research studies in relation to the intensity levels used in high vs. moderate exercise interventions.

### Desired Training Effects

Multiple desired training effects were sought in primary studies and reviewed in both of the systematic review studies. The most common intended training effect was improved walking speed using bodyweight supported treadmill training, used in 14 of the 17 (82%) studies (39, 40, 44–49). Improved CV fitness was used in 13 of the studies (76%) (39, 40, 44, 50–53). Changes to brain activity measured by an electroencephalogram (EEG) was reported in 5 (29%) of the 17 studies (47, 50, 54–56).

### Methods Used in Testing and Assessing Fitness in PwS

Intensity assessment was achieved using a graded exercise test (GXT) in 11 of the 15 (73%) primary research studies to obtain a predicted maximal oxygen consumption (VO_2_) and MHR measure (44, 45, 54, 57) and age-predicted MHR calculation (45, 48, 49, 54, 56). Two used maximal strength testing (55, 58), one used a home-based walking test and RPE to establish exercise effort (52), and one used age-predicted values for MHR and VO_2_ (56). Both of the review studies (39, 40) shared consistent findings with the primary studies in this review for the methods used to assess intensity level for interventions.

### Intensity Prescription and Within-Session Monitoring

All primary research studies used high-intensity exercise prescription with the effects captured over a maximum of 3 months (45). The two systematic review studies were consistent with this finding. Intensity progression within studies was prescribed most commonly using walking speeds (10 of 17 studies) (39, 44, 46–48, 50, 51, 56, 58). The RPE using the BORG 6-20 scale was used as a method of prescribing exercise intensity in 6 (29%) of the 17 studies (45, 47–49, 54, 56), while only one study used mixed methods for intensity prescription combining walking speed, percentage VO_2_, and recovery interval timings (39).

The monitoring of within-session intensity exercise using MHR was the most common method employed (10 studies) (44, 45, 47–49, 51, 54, 56, 57). The use of RPE and BORG 6-20 scales were also commonplace (nine studies) (44, 45, 47–49, 52–54, 56). Neither of the systematic review papers in this review reflected on the within-session monitoring methods during exercise interventions.

### Outcome Reporting

All of the primary research studies used outcome measures relating to body structure and function (as defined in the WHO ICF checklist) including VO_2_, HR, BP, blood lipids, blood biomarkers, interleukins, corticospinal excitability, and electromyography. Of these, Högg et al. (58) and Krawcyk et al. (52) used outcomes identified by Salter et al. (36) as reliable, valid, and responsive to change in PwS. Table 4 shows the outcome measures and how they line up with the WHO ICF constructs.

**Table 4:** Outcomes measure linked to WHO international classification of functioning, disability, and health constructs.

**First name author**	**Outcomes to measures used and relation to WHO ICF checklist**		
	**Body structure and function**	**Activity**	**Participation**
Aaron et al.		Walking speed on treadmill	
Abraha et al.	Maximum ventilatory threshold (VO_2_), Heart Rate (HR), Motor Evoked Potentials (MEP), Corticospinal Excitability (CSE), grip strength	***Box and block test***	
Boyne et al.	Exercise tolerance (completion of the 20 min session), VO_2_, HR	Walking speed on treadmill	
Carl et al.	Electrocardiogram (ECG)		
Crozier et al.	VO_2_, HR, MEP, Blood Pressure (BP)	***6-min walk test***, 10 MTW, ***Berg balance test***	
Gjellesvik et al.	VO_2_, BP, Blood profiles including High Density Lipoproteins (HDL), triglycerides, Glycated Haemoglobin (HbA1c), C-peptides		
Högg et al.	Grip strength, Motricity index, ***Fugl-Meyer assessment, modified ashworth scale***	Goal Attainment Scale (GAS)- specific to activity of an individual, ***Box and block test***	GAS- specific to participation of an individual
Krawcyk et al.	Endothelial function (plethysmography), hyperaemia index, HR and augmentation index, BP, multiple biomarkers (Pro-adrenomedullin, Pro-atrial natriuretic peptide, inter leukin 6, Tumour necrosis factor, ICAM-1 protein, VCAM-1 Biomarker, vascular endothelial growth factor. BMI. Multidimensional Fatigue Inventory (MFI-20 questionnaire), Major Depression Inventory (MDI), World Health Organisation Five well-being (WHO-5), Chronic stress Ull-meter, ***Montreal Cognitive Assessment***, Metabolic Equivalent of Task (MET) calculations from activity and HR measures,	Daily steps using accelerometer	Physical activity levels *via* Physical Activity Scale V2
Leggy et al.	VO_2_, MHR, oxygen cost walking from VO_2_	***6 MWT***	
Li et al.	EMG, TMS		
Luo et al.	VO_2_ and VO_2_ peak, pain VAS, injury rates	***6 MWT***, 10 MWT, Falls frequency,	
Madhavan et al.	Electromyography (EMG), Transcranial Magnetic Stimulation (TMS)	walking speed, 10 m walk	
Madhaven et al.	HR. BP	10-meter timed walk, ***6 MWT***	***Stroke Impact Scale (SIS)***
Mahtani et al.	HR, BP, Range of motion	Stepping symmetry, gait speed,	
Munari et al.	VO_2_, oxygen cost of walking, HP, BP,	10 MWT, ***6 MWT***, ***TUAG***	***SF-36 and SIS***
Nepveu et al.	TMS for CSE and Intra Cortical excitability, MVC,		
Urbin et al.	EMG, TMS, range of motion,		

*Outcomes in bold and underlined represent those specific to stroke from the ICF WHO Evidence Based Review of Stroke Rehabilitation (EBRSR) as identified and reviewed in (36)*.

*Bold represent outcomes from the ICF used in PwS*.

Five studies (51, 54–57) failed to use outcome measures related to activity, 12 (70%) used the 6 MWT, 5 (29%) used 10-meter walk test (10 MWT), and 4 (12%) used walking speeds obtained from the treadmill.

Four studies used outcome measures relating to participation (45, 48, 52, 58), of which two measures were recommended by Satler et al. (36), the Stroke Impact Scale and Short Form−36.

Six studies (45, 48–50, 53, 58) used outcome measures identified in Salter et al. (36), the most common of these being the 6 MWT. Only one of the systematic review studies (39) explored outcomes specific to PwS in exercise interventions. They also found that the 6 MWT was the most commonly used outcome and suggested this was due to the practicality and functional relevance for PwS.

Munair et al. (45) was the only study to explore the safety surrounding the use of high-intensity training intervention in stroke. Safety appeared to be supported in all studies as there was no mention of adverse events or dropouts of study participants. Neither of the systematic review studies explored safety issues.

## Discussions

This review has appraised a range of high-intensity interventions for PwS, which aim to increase CV fitness, improve muscle strength, increase functional capacity, or to increase brain activity.

### Homogeneity or Heterogeneity in Defining Intensity

All of the studies identified clear objectives and protocols of how they used high-intensity training. Munari et al. (45) was the only study that discussed and defined intensity and the role of intensity in interventions as the work rate, effort level, or metabolic demand of aerobic activity quantified by HR, rate of oxygen consumption, rating of perceived exertion, and/or walking speed. Despite various definitions of intensity in exercise interventions in non-clinical groups, there was no clear definition of intensity during exercise intervention in PwS. It remains unclear if defining intensity shares similarities or differences if the exercise intervention is aimed at a specific body system such as CV system compared with interventions aimed at improvements in a functional task such as walking speed.

### The Body System the Intervention Was Aimed At

Primary studies in this review and the two systematic review studies all had a key aim for exercise. This varied from improving CV fitness through changes to MHR or VO_2_ (44, 50), improving functional capacity through changes to walking speed (47, 48), and changes to brain activity *via* increased cortical firing rates (54). None of the studies sought to establish whether changes to a body system such as CV fitness actually resulted in improvement to function, or whether training a functional task such as walking would have differential impacts on the neurological, musculoskeletal, or CV systems.

Findings relating to intervention aims to support the Specific Adaption to Imposed Demands (SAID) principles identified in Sale et al. (59). These principles identify how the human body will adapt to any demand whether the stressor is biomechanical such as muscular, CV, or neurological. This can be observed in all of the primary studies that used treadmill training (44, 46–49, 53), where there is an identified improvement to walking function, but not necessarily changes in muscle strength, CV fitness, or motor potential.

### Methods Used in the Testing and Assessing Fitness in PwS

The assessment of intensity in studies ranged from the gold standard in healthy populations VO_2_ max testing (50) to graded exercise testing (44), RPE (53), and the talk test (52). Using graded exercise testing, percentage MHR from age-predicted value or obtained from GXT and RPE as assessment procedures are more practical and transferable to clinical practice. The studies were not consistent with their choice or reasoning for the assessment tool used. It was also unclear if using an assessment intervention such as percentage MHR or RPE using the BORG 6-20 scale showed any correlation with ability to achieve a percentage MHR in activities such as treadmill walking. No account was taken of other limiting factors e.g., lower limb strength rather than the CV demand of walking.

There was no clear consensus about an appropriate method for capturing baseline fitness of an individual. This was demonstrated by Munair et al. (45) who discussed intensity and its role in exercise prescription and how this needs to be specifically aimed at the appropriate body structure, functional task, or energy system the exercise intervention is being aimed at. They also discussed how other systems such as muscular strength or power may limit an individual reaching the desired level of intensity from a CV perspective.

### Protocols for Exercise-Intensity Prescription

A variety of methods were used to deliver an intensive intervention. These included achieving target percentage MHR or VO_2_ (53), percentage maximal walking speed (45), percentage of one repetition maximum (58), or adjusting recovery periods to a ratio or working intervals (44). There was some consistency in papers reviewed that working at or above 80–90% of MHR, VO_2_, or one repetition maximum of an individual classifies as a high-intensity intervention. It may be that a variety or combination of methods could be used to ensure sessions are high in intensity (39) and would be similar to periodization programs described by Lorenz et al. (60), where intensity of sessions is progressed in an undulating linear fashion allowing deloading or recuperation days. However, there was a lack of standardization for developing high-intensity training protocols. Different methods may create different outcomes or more specifically, certain methods used to create high-intensity sessions could be tailored depending on individuals pre-assessment fitness and ability findings. Eng et al. (13) and Flansbjer et al. (61) highlighted the importance of muscle strength in the performance of functional tasks and specifically correlations between lower extremity muscle strength and gait performance and how this relates to an increased perceived ease of participation during functional tasks.

No studies considered the long-term (more than 3 months) effects of short duration high-intensity training compared with the long-term effects of lower intensity longer duration training or higher volume training. In healthy adults, high-intensity strength-based training interventions and high-intensity anaerobic interventions demonstrate changes to body structure and function sharing similarities to aerobic training in relation to molecular signaling pathways (20). They also cause changes to muscle structure relevant to the stroke population such as improved pennation angle and sarcomere development. Future studies on exercise in stroke would benefit from investigating long term follow-up and combined interventions at various intensities to optimize protein synthesis and muscle architecture, potentially further enabling functional capacity in PwS.

### Within-Session Monitoring of Intensity

Within-session monitoring of individuals appeared appropriate to the intervention. Studies using a CV intervention such as treadmill training or cycling would typically use CV markers such as HR or VO_2_ calculations as a guide. RPE was one of the most commonly used methods with studies selecting the BORG 6-20 scale. Of the 17 included papers, only Krawcyk et al. (52) discussed the methods used to calibrate RPE (using the BORG 6-20 scale) and how this correlates to HR. None of the studies discussed the possible issues surrounding the inter-rater reliability of using a subjective perceptual scale, or if the perceived effort score is related to dyspnea or muscle fatigue.

The majority of the studies used a CV intervention to create changes to the CV system thereby increasing the functional capacity to walk further or more quickly. Some studies have used CV high-intensity training to investigate the benefits of brain activity by increasing cerebral blood flow (55, 56). Studies typically use electroencephalography (EEG) and transcranial magnetic stimulation (TMS) to evaluate the changes to brain activity. Both failed to evaluate if the intensity needed to create this change from a CV intervention was sufficient or appropriate for increasing brain activity as it was for increasing CV fitness.

### How Intensity Impacts of Changes in Exercise Intensity and How these Relate to Outcomes Within the Constructs of the WHO ICF Framework

All of the studies considered the use of outcome measures related to the body structure and function. This has been defined in the ICF checklist as the anatomical parts of the body and the physiological functions of body systems. These measures are keys for identifying the changes to CV fitness, muscle strength changes, and cortical excitability. While these changes may be of importance to elite athletes who are looking for the smallest of changes in competition, changes to functional activity and participation are thought to be more important to PwS (62). Outcome measures relating to activity and participation have been defined in the ICF checklist as the execution of a task or action by an individual i.e., stair climbing and the involvement of those tasks in real life situation i.e., climbing the stairs three times a day to use the toilet. This challenges the applicability of the studies for rehabilitation as their outcomes were not goal centered for PwS. None of the studies reviewed considered using this approach and despite the best efforts to ensure individuals are exercising at a specific intensity, it may be challenged that individual motivation may vary and could impact on their participation effort.

Stroke specific, validated outcome measures were used in eight of these studies. Salter et al. (36) assessed reliability, validity, and responsiveness of outcome measures in stroke. This review found functional testing such as the 6 MWT and Timed Up and Go (TUG) test were the most commonly used. Using outcome measures such as these, which may be more reliable, valid, and responsive and maybe more meaningful to the participant, might capture greater improvement from the intervention. None of the studies reported whether there was any education provided to the participants to help them understand the benefits of higher intensity training protocols.

All of the studies demonstrated a beneficial effect for high-intensity training on body systems, activities, and outcome measures. The limited use of functional outcome measures may be a factor in compliance and motivation in interventions. Högg et al. (58) used goal attainment scaling (GAS) of individuals to allow the individuals to select appropriate goals for fitness/activity/movement improvements. Sixty percent of the higher intensity and 55% of the moderate intensity group achieved their participation-specific GAS outcomes. All groups increased in grip strength and most experienced no changes to spasticity.

While there was an identified need to use a harness in walking intervention as a safety precaution, there was minimal discussion about safety and the need to tailor exercise to meet the specific needs of each individual with stroke, and there was no record of how the needs of PwS were addressed when there were issues.

### Comparison to Previous Literature

Nichols et al. (63) report that exercise intensity in cardiac rehabilitation programs can be suboptimal. This may limit potential intervention benefits on neuroplasticity, strength, and CV fitness in programs treating PwS. Neurological training specifically lacks an appropriate methodology to measure intensity during skill training (19) and as a consequence, potential neuroplastic gains made through skill or sensory-motor training, strength training, and CV training in individuals may not be optimal.

Due to the high-intensity nature of the studies used in this review and lack of short term follow-up, it remains unclear if increasing the intensity of exercise provides any significant long-term physiological, physical, or psychological benefits to PwS over and above high volume-low-intensity training. None of the studies in this review identified how individuals need the sufficient support systems such as the neurological function and muscle power to participate in varied CV-based interventions, something that is taken for granted in non-clinical groups or clinical groups who do not have significant physical impairments. Furthermore, rigorous assessments of all body systems would allow for an appropriate selection of assessment tools to establish tailored intensity levels or thresholds for the desired body system. Methods of monitoring the intensity of the session specific to the intervention and using specific and sensitive outcome measures to detect changes at all levels of the ICF is key. More specifically, the need to identify benefits tailored to an individual.

This review has identified that changes to mood and quality of life can be related to the physiological changes brought about by the exercise component. None of the reviews acknowledged the potential social benefits of exercise participation, which might bring about improvements to mood and quality of life (57).

This review also did not identify how additional strength gains may not be associated with further improvement in an activity (5). Strengthening beyond the functional needs of an individual may be of value for establishing a functional reserve rather than further improvement in current performance at a functional activity. This may also be the case with neurological/skill training and CV-based training (64).

## Conclusions

This review has explored the use of intensity in exercise training intervention for PwS, and how this varies depending on the desired effect on the body system or task-specific activity. The tools most employed to gauge exercise intensity and that can be translated to clinical practice for monitoring intensity are MHR and BORG RPE. Despite this, there is a lack of consensus about how to define exercise intensity across CV, muscular strength, neurological, and functional skill training, and how this is applied in a meaningful way to PwS to optimize the benefits. A clear understanding of intensity is essential to focus the desired training effect required in exercise interventions and improve the prescription of intensity by therapists and exercise prescribers. More focus on the desired effect would allow the appropriate intensity training methods to be selected and consideration given to whether longer duration, moderate intensity training should be combined with higher intensity training for optimal benefits. A thorough understanding of the needs of the PwS, specifically the multidimensional issues they present with, is required in order to tailor the intensity, type of exercise, and methods of training. Although not covered in this review, the need for education related to the intervention needs to be considered when selecting outcome measures. This includes the exercise desired effects at a physiological level and how this can be used to improve meaningful outcomes such as skill reacquisition. This can then be used to allow PwS to see how these benefits can impact on the achievement of everyday tasks and furthermore into the reintegration into social participation.

Finally, we need a better understanding of the timescales required for exercise interventions to make the desired changes in PwS. It is unlikely that single bout interventions are able to provide a meaningful snapshot of the actual benefits of varied exercise-intensity interventions. Additionally, if there are superior health-related benefits with higher intensity training, further consideration is need about the effect on long-term adherence compared to lower intensity exercise interventions in PwS.

## Author Contributions

GC, KS, AA, and CS: conceived and designed the study. KS, CS, and AA: critical review of paper. GC: writing of paper, analysis, interpretation of findings, and conducting literature searches. GC and KS: piloting data collection and quality measure. GC, CS, and KS: inclusion and exclusion process. All authors contributed to the article and approved the submitted version.

## Funding

GC was funded by Health Education England (HEE)/National Institute for Health Research (NIHR) on a Pre Doctoral Research Fellow (NIHR300426).

## Author Disclaimer

The views expressed in this publication are those of the author(s) and not necessarily those of the NIHR, Sheffield Teaching Hospitals NHS FT, NHS, Sheffield Hallam University or the UK Department of Health and Social Care.

## Conflict of Interest

The authors declare that the research was conducted in the absence of any commercial or financial relationships that could be construed as a potential conflict of interest.

## Publisher's Note

All claims expressed in this article are solely those of the authors and do not necessarily represent those of their affiliated organizations, or those of the publisher, the editors and the reviewers. Any product that may be evaluated in this article, or claim that may be made by its manufacturer, is not guaranteed or endorsed by the publisher.
